# Secret Remedies and Branded Products—V

**Published:** 1908-04-18

**Authors:** 


### Therapeutics.

### SECRET REMEDIES AND BRANDED PRODUCTS—V.

[It is the object of these articles to show how departure from rational prescribing has fostered and encouraged a system which is open to many grievous abuses, of which self-medication is not the least. The writer appreciates the fact that many valuable preparations are sold under proprietary and protected names, and wishes it clearly to be understood that products which are recognised by medical practitioners on account of their real utility are in no way called in question.]

At this point it may be useful to consider to what extent the trend of modern prescription writing is in favour of secret remedies and branded products, for by this means one can form an idea of the degree to which the nostrum traffic is aided and abetted by medical practitioners.

As a guardian of the public health, it is the duty of the physician to expose and to destroy any system which is a source of danger to health, and therefore the distribution of quack medicines is an evil which he must do all in his power to check. Although the whole question is one which demands the serious consideration of the profession, there is one particular phase of it which concerns him more intimately than any other, namely the distribution of those products, which are sold for the most part through the intermediary of a medical prescription. In a measure the nostrum advertised to the public and the nostrum advertised to the physician are based on the same principles; special virtues are claimed for both, and both are the private property of commercial persons or firms, whose interest it is to sell their particular product to the exclusion of others; and in many cases both are produced by pretentious quacks. The "patent" pill, whose virtues are proclaimed in mendacious leaflets, distributed from door to door, is a very near relative of many a proprietary product, whose alleged therapeutical action is described by high-sounding phrases —generally translated from the German—contained in neat little pamphlets sent through the post to every address on the medical register with offers of free samples.

From a careful examination of the prescription books of a number of pharmaceutical chemists in different parts of London, it appears that out of every 500 prescriptions copied, about 105 consist wholly or in part of branded products, a large proportion of which are compounds whose composition is known only to the makers. A correct estimate of the extent to which proprietary articles are prescribed cannot, however, be obtained from prescription books, for, whereas formerly it was the custom of every pharmacist to copy each prescription which he dispensed, such is not now the invariable practice.

On the authority of pharmacists of high standing it can be stated that it is a common occurrence for clients to ask for the branded product ordered by the physician without handing in the prescription. As records are not kept of such transactions, it cannot be stated precisely to what extent this custom is prevalent, but there is evidence that the percentage of prescriptions containing proprietary articles is much higher than that shown by the records. It not unfrequently happens that a patient, instead of handing the prescription to the pharmacist, reads from it the name of the proprietary article prescribed. Such a compound is handed over in the original package, and henceforth that patient becomes a keen competitor of the physician who prescribed the ready packed product. He will probably meet an acquaintance who contemplates consulting the same practitioner for what he thinks is a similar disease, and the man with the prescription will say, "You needn't go to see him, get a box of So-and-so's cachets of compound something or other, that's what he prescribed for me." Such advice is very common, and is all too frequently adopted with disastrous results, for the person who takes the advice may be suffering from a totally different complaint from the person who gave it.

Sometimes the physician loses the confidence of his patient by prescribing a proprietary article, as the following occurrence, which is vouched for by a pharmacist in the West End of London, shows. A client handed in a prescription written by a consultant, and, as is usually the case, asked how long it would take to prepare it. The chemist looked at the prescription and saw written the name of a widely advertised saline preparation, a bottle of which he at once handed to his client. The latter, at first, refused to believe that the consultant had prescribed such an article, for in his own words he had "already taken five bottles before going to see the doctor!"

However, it is an ill wind that blows nobody any good, and if the practitioner and the patient suffer because of the prescriber's leaning towards ready packed products, there is one person who reaps the benefit—and that is the proprietor of the prescribed product.

